# Repetitive Transcranial Magnetic Stimulation in Migraine: Clinical Outcomes and Neurobiological Mechanisms—A Systematic Review

**DOI:** 10.3390/neurolint18050080

**Published:** 2026-04-27

**Authors:** Robert Constantin Zgarbura, Leea Cristescu Rizea, Madalin Dinca, Alexandru Pavel, Oana-Andreea Parliteanu, Jari Sabri, Catalina Tudose

**Affiliations:** 1 Psychiatry Department, University of Medicine and Pharmacy “Carol Davila”, 050474 Bucharest, Romania; robert-constantin.zgarbura@drd.umfcd.ro (R.C.Z.); helga-leea.cristescu2022@stud.umfcd.ro (L.C.R.); madalin-marius.dinca2022@stud.umfcd.ro (M.D.); catalina.tudose@umfcd.ro (C.T.); 2 FutureMeds, 031441 Bucharest, Romania; 3 PAX Clinic, 020951 Bucharest, Romania; 4 Diabetes Department, Pneumology Institute “Marius Nasta”, 050159 Bucharest, Romania; 5 Faculty of Medicine, University of Medicine and Pharmacy “Titu Maiorescu”, 040051 Bucharest, Romania; 6 Faculty of Physical Education and Sports, “Spiru Haret” University, 041905 Bucharest, Romania; jari.sabri@spiruharet.ro

**Keywords:** rTMS, migraine, headache, systematic review, sham

## Abstract

Background: Migraine is a highly prevalent neurological disorder associated with substantial disability and socioeconomic burden. Although pharmacological therapies remain the mainstay of treatment, their effectiveness may be limited by incomplete response and adverse effects. Repetitive transcranial magnetic stimulation (rTMS) has emerged as a non-invasive neuromodulatory technique that may modulate cortical excitability and pain-processing networks involved in migraine pathophysiology. This systematic review aimed to evaluate the current evidence regarding the efficacy and safety of rTMS compared with sham stimulation in individuals with migraine. Methods: A systematic search was conducted in PubMed (MEDLINE), PsycNet, and Ovid (including MEDLINE and Embase) from database inception to December 2025 in accordance with PRISMA 2020 guidelines. Studies investigating rTMS in adults with migraine and including a sham comparator were eligible for inclusion. Data regarding study design, participant characteristics, rTMS parameters, outcomes, and adverse events were extracted using a predefined template. Risk of bias was assessed using the Cochrane Risk of Bias 2 tool. Results: Seven studies comprising a total of 301 participants were included. Most trials evaluated high-frequency rTMS targeting the dorsolateral prefrontal cortex. Across studies, rTMS was generally associated with reductions in migraine frequency and severity compared with sham stimulation, although results varied depending on stimulation parameters and study design. Treatment was consistently well tolerated, with only mild and transient adverse effects reported. However, considerable heterogeneity was observed in diagnostic criteria, stimulation protocols, outcome measures, and follow-up duration. Conclusions: Preliminary evidence suggests that rTMS may represent a promising and well-tolerated neuromodulatory approach for migraine management. Nevertheless, methodological variability, limited sample sizes, and concerns regarding risk of bias restrict definitive conclusions. Larger randomized controlled trials with standardized protocols and longer follow-up periods are needed to clarify the clinical role of rTMS in migraine treatment.

## 1. Introduction

Migraine is a highly prevalent neurological disorder characterized by recurrent attacks of moderate-to-severe headache, typically unilateral and pulsating in quality, often accompanied by nausea, photophobia, and phonophobia [1]. According to the International Classification of Headache Disorders, third edition (ICHD-3), migraine exists on a clinical spectrum ranging from episodic migraine (EM), defined by fewer than 15 headache days per month, to chronic migraine (CM), characterized by 15 or more headache days per month for at least three months, of which at least eight fulfill migraine criteria [2]. This distinction carries significant prognostic and therapeutic implications, as chronic migraine is associated with greater disability, higher healthcare utilization, and a considerably more complex treatment landscape than its episodic counterpart.

According to the World Health Organization, migraine ranks among the top 20 most disabling diseases worldwide [3]. It affects approximately 18% of women and 6% of men between the ages of 25 and 55, with prevalence gradually declining after the age of 40 [4,5].

Beyond the pain itself, migraine is associated with a wide range of comorbidities, including stress, sleep disturbances, depression, and even increased risk of suicidal behavior [6]. The disorder also imposes a substantial socioeconomic burden due to healthcare costs and reduced productivity [7].

Non-pharmacological approaches such as mindfulness and behavioral therapies have demonstrated benefit in migraine management [8], providing patients with non-invasive options for symptom control. Pharmacological management of migraine encompasses two complementary strategies: acute (abortive) treatment and preventive (prophylactic) treatment, each serving distinct clinical roles. Acute therapies aim to abort or relieve individual migraine attacks as they occur. Non-steroidal anti-inflammatory drugs (NSAIDs) and acetaminophen represent first-line options for mild-to-moderate attacks, though NSAIDs carry risks of gastrointestinal complications, increased bleeding risk, and medication-overuse headache with prolonged use [9]. Triptans (serotonin 5-HT1B/1D receptor agonists) and ergots are more migraine-specific acute agents, effective for moderate-to-severe attacks, but may be poorly tolerated and carry cardiovascular contraindications [9,10]. More recently, gepants—calcitonin gene-related peptide (CGRP) receptor antagonists, including ubrogepant, rimegepant, and atogepant—have emerged as effective acute agents without vasoconstrictive risk and, in the case of rimegepant and atogepant, hold additional regulatory approval for preventive use [11].

Preventive therapies, in contrast, are aimed at reducing the frequency, severity, and duration of migraine attacks over time and are indicated when attacks are frequent, disabling, or inadequately controlled with acute treatment. Traditional preventive agents include antiepileptic drugs (topiramate, valproate), antidepressants (amitriptyline, venlafaxine), and antihypertensive agents (propranolol, candesartan) [9]. OnabotulinumtoxinA has demonstrated efficacy specifically in chronic migraine and is approved for this indication [12]. More recently, monoclonal antibodies targeting CGRP or its receptor—erenumab, fremanezumab, galcanezumab, and eptinezumab—have substantially advanced the preventive treatment landscape, providing migraine-specific, well-tolerated options with once-monthly or quarterly dosing schedules [13]. Despite this expanding armamentarium, a significant proportion of patients do not achieve adequate response or experience intolerable adverse effects, underscoring the continued need for effective non-pharmacological alternatives.

Given these limitations, interest in neuromodulatory approaches has grown considerably. Transcranial magnetic stimulation (TMS) is a non-invasive neurostimulation technique based on electromagnetic induction, whereby a rapidly changing magnetic field generates a secondary electric field in the underlying cortical tissue, resulting in transient neuronal depolarization [14]. When delivered repetitively (rTMS), it can modulate cortical excitability in a sustained manner, with effects extending well beyond the stimulation period itself. These neuroplastic changes are thought to be mediated by long-term potentiation (LTP)- and long-term depression (LTD)-like mechanisms [15]. Crucially, the direction of cortical excitability changes depends primarily on stimulation frequency: low-frequency rTMS (≤1 Hz) is generally associated with inhibitory, suppressive effects, whereas high-frequency rTMS (≥5 Hz, most commonly 10–20 Hz) tends to be facilitatory and excitability-enhancing [15].

In migraine research, the dorsolateral prefrontal cortex (DLPFC) has emerged as a primary stimulation target. The DLPFC plays a central role in descending pain modulation through its projections to the periaqueductal gray (PAG) and its extensive connections within frontal-limbic and thalamocortical networks implicated in migraine pathophysiology [16,17]. Stimulation of the DLPFC is hypothesized to enhance top-down inhibitory control over pain processing, thereby counteracting the cortical hyperexcitability that characterizes migraine interictal states. Alternative cortical targets investigated in the literature include the vertex and the motor cortex. Typical rTMS protocols in migraine research have employed 600–1600 pulses per session at intensities of 70–90% of the resting motor threshold (RMT), administered across 3 to 23 sessions spanning several days to weeks [15]. Several clinical studies have reported reductions in migraine days and headache severity following rTMS treatment, suggesting it may represent a promising and well-tolerated therapeutic alternative with fewer systemic adverse effects than pharmacological therapies [18,19].

However, despite statistically significant findings in some trials, the strength of evidence regarding the clinical applicability of rTMS remains uncertain due to considerable heterogeneity in study designs. Variations in patient selection, stimulation parameters, cortical targets, and follow-up duration make it difficult to determine optimal treatment protocols. The aim of this systematic review is therefore to evaluate the current evidence regarding the efficacy and safety of rTMS compared with sham stimulation in adults with migraine, and to identify key methodological challenges that future research should address.

## 2. Materials and Methods

This systematic review was conducted in accordance with the Preferred Reporting Items for Systematic Reviews and Meta-Analyses (PRISMA) 2020 guidelines [20]. This systematic review was registered in the Open Science Framework (OSF) (registration DOI: 10.17605/OSF.IO/P58WT). Additional details were also provided in Table S2: PRISMA Checklist.

### 2.1. Search Strategy

A comprehensive literature search was performed to identify all studies investigating the effects of repetitive transcranial magnetic stimulation (rTMS) in individuals with migraine. The following databases were searched from inception to December 2025: PubMed (MEDLINE), PsycNet, and Ovid (including Ovid MEDLINE and Ovid Embase).

The search strategy combined controlled vocabulary terms (e.g., MeSH and database-specific thesaurus terms) with free-text keywords related to “repetitive transcranial magnetic stimulation”, “rTMS”, and “migraine”. Full search strings for each database are provided in Supplementary Table S1. Only articles written in English and reporting studies performed on adult participants were eligible. No restrictions were applied regarding publication date. Reference lists of included articles and relevant reviews were manually screened to identify additional eligible studies.

### 2.2. Eligibility Criteria

Studies were considered eligible if they met all of the following criteria:

Population: Adults (≥18 years) diagnosed with migraine, regardless of subtype, according to recognized diagnostic criteria. Studies were eligible regardless of the specific version of criteria applied—including IHS, ICHD-2, ICHD-3 beta, or ICHD-3—reflecting the temporal span of published trials. Variability in diagnostic criteria across included studies was systematically recorded during data extraction and is addressed as a source of heterogeneity in the interpretation of results.

Intervention: Repetitive transcranial magnetic stimulation (rTMS), irrespective of stimulation parameters (frequency, target region, coil type, intensity, number of pulses, or treatment duration).

Comparator: Sham stimulation; studies allowing stable concomitant migraine treatments were eligible.

Outcomes: Migraine-related outcomes, including but not limited to headache frequency, intensity, duration, migraine days, disability indices (e.g., MIDAS) or neurophysiological measures associated with migraine.

Study design: Randomized controlled trials.

Exclusion criteria were: prospective or retrospective observational studies, open-label trials, case reports, case series with fewer than five participants, conference abstracts without full-text availability, review articles, editorials, animal studies, and studies not reporting migraine-specific outcomes separately.

### 2.3. Study Selection

All identified records were exported into a reference management software, and duplicate entries were removed.

Two reviewers (M.D. and L.C.) independently screened titles and abstracts for eligibility. Potentially relevant articles underwent full-text review. Discrepancies at any stage were resolved through discussion. If consensus was not reached, two additional reviewers (R.Z. and A.P.) were consulted, and agreement was achieved through panel discussion.

### 2.4. Data Extraction

Data were extracted independently by two reviewers using a predefined, standardized data extraction form.

The following information was collected: study characteristics (authors, year, country, design), sample size and demographic characteristics, diagnostic criteria for migraine, rTMS protocol parameters (target region, stimulation frequency, intensity relative to motor threshold, number of pulses per session, coil type, number and duration of sessions), concomitant migraine treatments, outcome measures and assessment time points, main efficacy findings and reported adverse events.

When necessary, corresponding authors were contacted to clarify incomplete or unclear data.

### 2.5. Risk of Bias Assessment

Risk of bias was assessed independently by two reviewers. Randomized controlled trials were evaluated using the Cochrane Risk of Bias 2 (RoB 2) tool [21], assessing bias across the following domains: (1) randomization process, (2) deviations from intended interventions, (3) missing outcome data, (4) measurement of the outcome, and (5) selection of the reported result.

Given the heterogeneity of headache outcome measures across studies, risk of bias judgments were performed for the primary headache-related outcome as defined by each individual study at the earliest post-treatment time point. Disagreements were resolved through discussion until consensus was reached.

## 3. Results

**Figure 1:**
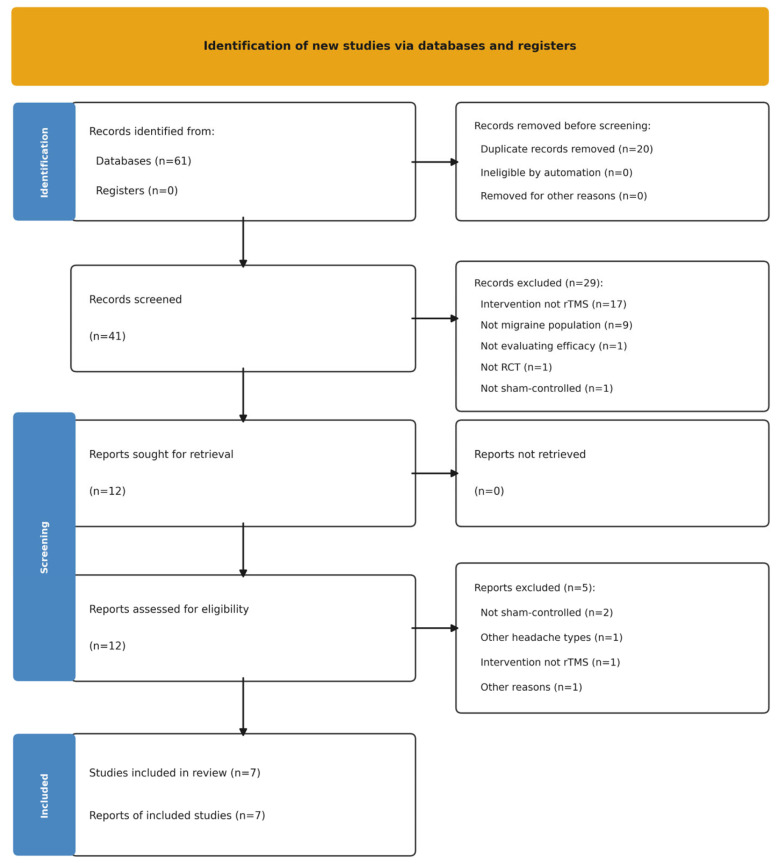
PRISMA Flow Diagram.

The initial database search identified 61 records. After removal of duplicates (n = 20), 41 records underwent title and abstract screening, of which 29 were excluded for not meeting the eligibility criteria. The remaining 12 reports proceeded to full-text review, where a further 5 were excluded due to ineligibility. Ultimately, seven studies met all inclusion criteria and none of the exclusion criteria and were included in the systematic review. The study selection process is presented in Figure 1.

### 3.1. Study Characteristics and Protocol Parameters

The seven included studies were published between 2004 and 2025 and enrolled a total of 301 participants, with individual sample sizes ranging from 11 (Brighina et al., 2004) [22] to 100 (Misra et al., 2013) [23]. Three studies were conducted in India [23,24,25], and one each in China [26], Brazil [27], Germany [19], and Italy [22].

Diagnostic criteria varied considerably across studies. Earlier trials—Brighina et al. (2004) [22], Teepker et al. (2010) [19], Misra et al. (2013) [23], and Kalita et al. (2016) [25]—defined eligibility based on attack frequency thresholds (typically ≥4 migraine attacks or headache days per month), without reference to formal classification systems. More recent studies—Conforto et al. (2014) [27], Kumar et al. (2020) [24], and Song et al. (2025) [26]—applied formal ICHD-based diagnostic criteria. This variability in diagnostic standards reflects the evolution of classification systems over the study period and represents an important source of cross-study heterogeneity.

Stimulation parameters differed substantially across trials. Stimulation frequency ranged from 1 Hz (Teepker et al.) [19] to 20 Hz (Brighina et al.; Song et al.) [22,26], with the majority of protocols employing 10 Hz. Five of seven studies targeted the left DLPFC [22,24,26,27], one targeted the left frontal cortex [23,25], and one targeted the vertex [19]. Stimulation intensity ranged from 70% to 90% of the resting motor threshold (RMT). Pulses delivered per session ranged from 600 to 1600, and the total number of treatment sessions ranged from 3 to 23. This wide variation in stimulation architecture precluded the derivation of a meaningful optimal protocol from the existing data and substantially complicated cross-study comparisons.

### 3.2. Individual Study Summaries

Song et al. (2025) [26] conducted a randomized, double-blind, sham-controlled trial in 28 medication-naïve patients with migraine (ICHD-3; with or without aura) in China. High-frequency rTMS (20 Hz, 700 pulses/session) was applied to the left DLPFC daily for 14 consecutive days. Active stimulation was associated with significant reductions in monthly migraine days (MMD) and VAS-rated headache severity relative to baseline, while the sham group showed no meaningful change. This study was the only one in the present review rated at low risk of bias across all RoB 2 domains.

Misra et al. (2013) [23] enrolled the largest sample (n = 100) in a randomized, double-blind design. Left frontal cortex rTMS at 10 Hz (70% MT, 600 pulses/session) was administered across three alternate-day sessions. Active treatment produced significant reductions in headache frequency and VAS severity, with more than 50% of participants in the active group classified as responders (>50% VAS improvement), compared with a smaller proportion in the sham group. The study was rated as having some concerns, primarily related to outcome measurement.

Conforto et al. (2014) [27] conducted a proof-of-principle randomized trial in 18 patients with chronic migraine already receiving stable pharmacological prophylaxis. Despite an intensive protocol—10 Hz, 1600 pulses/session over 23 sessions targeting the left DLPFC—active rTMS did not demonstrate superiority over sham for either headache days or MIDAS disability scores. Notably, the sham group showed a statistically significant reduction in both outcomes, possibly reflecting strong placebo effects or spontaneous fluctuation in chronic migraine course. The study was rated as having some concerns.

Kumar et al. (2020) [24] employed a neuronavigation-guided protocol (10 Hz, 70% RMT, 600 pulses/session, 10 sessions over the left DLPFC) in 20 patients with ICHD-3 chronic migraine. Active rTMS was associated with significant reductions in headache frequency at a 3-month follow-up compared with sham, though no between-group difference was observed for MIDAS scores. The study was rated as having some concerns.

Brighina et al. (2004) [22], in the earliest and smallest included trial (n = 11; 6 active, 5 sham), applied 20 Hz rTMS (90% MT, 10 trains/session) to the left DLPFC in patients with chronic migraine. Active stimulation produced significant and sustained reductions in attack frequency, headache index, and rescue medication use at 1–2 months of follow-up, with no significant change in the sham group. Despite the small sample size, the directional findings were consistent with results from other high-frequency DLPFC protocols. The study was rated as having some concerns.

Kalita et al. (2016) [25] conducted a partially sham-controlled trial in 98 participants (52 active, 46 sham), comparing one versus three sessions of 10 Hz rTMS to the left frontal cortex in patients with chronic migraine and tension-type headache. The three-session active rTMS condition produced a >50% VAS improvement in 78.6% of participants, compared with 34.2% in the sham group—the largest between-group difference reported across all included studies. However, the partially sham-controlled design and concerns about outcome assessment methodology resulted in a high risk of bias rating for this trial.

Teepker et al. (2010) [19] applied a distinctly different approach—low-frequency 1 Hz rTMS (1000 pulses/day, 5 consecutive days) targeting the vertex—in 27 patients with episodic migraine. While within-group improvements were observed in the active arm, no statistically significant between-group differences were found for migraine attack frequency, headache days, or total headache hours. Several participants in the active group discontinued treatment due to scalp discomfort. The study was rated as having some concerns.

### 3.3. Outcome Heterogeneity and Overall Evidence

Across the seven included studies, outcome measures encompassed migraine frequency or number of migraine days (all studies), VAS-rated pain intensity (five studies), and disability assessed via MIDAS (two studies). Follow-up durations ranged from 4 weeks (Conforto et al., 2014) [27] to 12 weeks (Kumar et al., 2020) [24], with a one-month post-treatment assessment most commonly employed. The diversity of outcome instruments, the absence of a standardized primary endpoint, and the wide variation in follow-up duration substantially limited cross-study comparability. Furthermore, standardized effect sizes and confidence intervals for between-group comparisons were not consistently reported, constraining the ability to characterize the magnitude of treatment effects. These factors collectively precluded quantitative meta-analytic synthesis.

Taken together, preliminary evidence suggests a trend toward benefit with active rTMS—particularly high-frequency protocols targeting the DLPFC—compared with sham stimulation. However, given the substantial heterogeneity in protocols, diagnostic criteria, outcome measures, and sample sizes, as well as a risk of bias profile ranging from some concerns to high in six of seven studies, these findings should be regarded as exploratory. The currently available evidence does not support definitive conclusions regarding the efficacy of rTMS in migraine.

A detailed summary of study characteristics and findings is presented in Table 1.

## 4. Discussion

Migraine remains a major global public health concern, given its high prevalence and substantial contribution to the global burden of disease [28]. The search for effective and well-tolerated treatment alternatives therefore remains an important priority. Repetitive transcranial magnetic stimulation (rTMS) represents a promising neuromodulatory approach, acting through the depolarization of myelinated axons and modulation of connectivity within cortical pain-processing networks [29]. By influencing cortical excitability, rTMS may help counteract the neural dysregulation observed in both episodic and chronic migraine. The findings of the present systematic review suggest a trend toward benefit with active rTMS over sham stimulation, particularly with high-frequency DLPFC protocols; however, the strength of this evidence is modest, and these observations must be interpreted as preliminary.

With the exception of Conforto et al. (2014) [27], most included studies reported improvements in headache-related outcomes compared with sham stimulation. A consistent pattern across trials was that protocols employing high-frequency stimulation of the DLPFC tended to report more robust reductions in migraine frequency and severity. In contrast, the study applying vertex stimulation at 1 Hz (Teepker et al.) produced no statistically significant between-group differences, reinforcing the notion that both cortical target and stimulation frequency are critical determinants of therapeutic response.

The potential mechanisms underlying the effects of rTMS in migraine are likely multifactorial. Stimulation of the DLPFC may influence migraine symptoms through modulation of fronto-limbic networks involved in pain perception and affective processing, as well as through downstream effects on thalamocortical pathways implicated in migraine pathophysiology [17]. By altering the balance of cortical excitability and facilitating top-down modulation of nociceptive signaling, rTMS may contribute to the normalization of dysfunctional neural networks associated with migraine. The divergent outcomes between high-frequency and low-frequency protocols may further reflect distinct neuroplastic mechanisms: high-frequency rTMS promotes LTP-like cortical facilitation and may enhance descending inhibitory pathways, whereas low-frequency protocols tend to induce LTD-like inhibition, with less predictable downstream effects on pain modulation networks [15].

### 4.1. Rationale for Exclusion of Meta-Analysis

Although a meta-analytic synthesis was not performed in the present review, the reasons for this decision warrant explicit discussion. The substantial heterogeneity observed across included studies—spanning differences in stimulation frequency (1–20 Hz), cortical targets, pulse delivery parameters, session counts (3–23), diagnostic criteria, and outcome measures—rendered quantitative pooling clinically inappropriate. In the presence of such methodological diversity, pooled effect estimates would risk being difficult to interpret and potentially misleading. Furthermore, several included studies did not report standardized effect sizes or confidence intervals for between-group comparisons, precluding the derivation of reliable pooled estimates. Future systematic reviews incorporating more homogeneous trial populations and standardized protocols may be better positioned to perform meta-analyses.

### 4.2. Influence of Risk of Bias on Results

The risk of bias assessment revealed important concerns that must be considered when interpreting the present findings. Only one study (Song et al., 2025) [26] was rated at low risk of bias; five studies received some concerns ratings, and one (Kalita et al., 2016) [25] was rated at high risk of bias. The predominant sources of concern included the domain of outcome measurement—particularly regarding the integrity of blinding of outcome assessors—potential deviations from intended interventions, and the risk of selective outcome reporting. In studies where blinding may have been inadequate, the likely direction of bias would favor overestimation of treatment effects in the active rTMS arm. Notably, Kalita et al. [25]—the study rated at high risk of bias—simultaneously reported the largest between-group treatment difference of any included trial, illustrating how methodological limitations can potentially inflate apparent efficacy estimates. The preponderance of studies with at least some concerns regarding bias reinforces the need for cautious interpretation of the current evidence base and highlights the imperative for methodologically rigorous, adequately blinded trials.

### 4.3. Methodological Heterogeneity and Clinical Implications

Despite the generally favorable direction of findings in the included studies, interpretation of the available evidence is substantially complicated by methodological heterogeneity. Differences were observed in follow-up duration, diagnostic criteria, stimulation parameters, and outcome measures. While more recent studies applied formal ICHD-based diagnostic criteria, earlier studies relied primarily on attack frequency thresholds. Such variability may influence patient selection and disease characterization, thereby affecting treatment response and limiting comparability across trials.

In addition to diagnostic variability, the stimulation protocols themselves differed considerably, including frequency (ranging from 1 to 20 Hz), cortical targets (DLPFC, frontal cortex, or vertex), number of treatment sessions, and total pulse delivery. These differences make it difficult to determine the optimal stimulation protocol and may partially explain the variability in treatment outcomes reported in the literature.

Outcome assessment also lacked uniformity. The reviewed studies employed different scales to evaluate treatment response, including headache frequency measures, VAS ratings, and MIDAS scores. Although each of these tools provides clinically relevant information, the absence of a consistent primary outcome measure impairs evidence synthesis. Future trials would benefit from adopting standardized, multidimensional outcome frameworks that incorporate both frequency-based and disability-based measures, and from reporting standardized effect sizes and confidence intervals to facilitate future meta-analytic work.

Across the included trials, rTMS was generally reported to be a safe and well-tolerated intervention, with only mild and transient adverse effects described. Most reported side effects consisted of scalp discomfort, transient headache, or mild fatigue. This favorable safety profile is particularly relevant given the limitations and potential adverse effects associated with many pharmacological migraine treatments.

From a clinical perspective, rTMS may represent a potential adjunctive therapeutic option for patients with migraine who do not respond adequately to pharmacological therapy or who experience medication-related adverse effects. However, the current evidence base is limited by relatively small sample sizes, heterogeneous protocols, and short follow-up durations. It also remains unclear which subgroups of patients—such as those with episodic versus chronic migraine, with or without aura, or who have failed specific pharmacological classes—may derive the greatest benefit from neuromodulatory treatment. These uncertainties should temper clinical enthusiasm pending results from larger and more rigorously designed trials.

Future research should therefore focus on conducting larger, adequately powered randomized trials with longer follow-up periods and standardized outcome measures. In addition, efforts should be directed toward identifying optimal stimulation parameters and clarifying which patient characteristics may predict treatment response. Standardized reporting of between-group effect sizes and confidence intervals should be considered a minimum requirement for future trials in this area.

### 4.4. Limitations

This systematic review has several limitations. First, the number of available randomized controlled trials investigating rTMS in migraine remains relatively small, and most studies included limited sample sizes, reducing statistical power and the generalizability of findings. Second, the substantial heterogeneity in stimulation protocols, patient populations, and outcome measures restricted direct comparison across trials and prevented quantitative synthesis through meta-analysis. Third, the relatively short follow-up periods reported in most studies limit conclusions regarding the long-term efficacy and safety of rTMS in migraine prevention. Fourth, the risk of bias assessment revealed that six of seven studies had at least some methodological concerns, and the absence of standardized effect size reporting further constrains the precision with which treatment effects can be characterized. Collectively, these limitations underscore the preliminary and exploratory nature of the available evidence.

## 5. Conclusions

The available preliminary evidence suggests that repetitive transcranial magnetic stimulation—particularly high-frequency protocols targeting the dorsolateral prefrontal cortex—may reduce migraine frequency and severity while maintaining a favorable tolerability profile. However, the current body of evidence is limited by substantial methodological heterogeneity, small sample sizes, variability in stimulation protocols, short follow-up durations, and concerns regarding risk of bias in the majority of included studies. Definitive conclusions regarding the clinical efficacy of rTMS in migraine cannot be drawn from the existing literature. Future large-scale, prospectively registered randomized controlled trials with standardized stimulation protocols, uniform outcome measures, and longer follow-up periods are essential to better define the clinical role of rTMS in migraine management and to identify patient subgroups most likely to benefit from this neuromodulatory approach.

## Figures and Tables

**Table 1:** Detailed characteristics of the included studies.

Author (Year)	Country	Study Design	Total (n)	Intervention/Sham (n)	Age (Years)	Sex (% Female)	Comparator	Key Inclusion Criteria	rTMS Protocol	Coil Type	Concomitant Migraine Treatment	Headache Outcomes	Follow-Up	Main Findings (Intervention)	Main Findings (Sham)	Conclusion	Adverse events	RoB
Song et al., 2025 [26]	China	Randomized, double-blind, sham-controlled	28	14/14	37.31 ± 6.40	62.9%	Sham	Adults 18–65 y; migraine with/without aura (ICHD-3); medication-naïve	Left DLPFC; 20 Hz; 90% RMT; 700 pulses/session; daily ×14 days	Figure-of-eight (70 mm)	None	Monthly migraine days (MMD); VAS severity	1 month	Reduction in MMD and headache severity compared with baseline	No meaningful change from baseline	HF-rTMS over left DLPFC reduced migraine burden	None reported	Low
Misra et al., 2013 [23]	India	Randomized, double-blind, sham-controlled	100	50/50	35.34 ± 10.22	88%	Sham	≥4 migraine attacks/month for ≥3 months	Left frontal cortex; 10 Hz; 70% MT; 600 pulses/session; 3 sessions on alternate days	Figure-of-eight (7 cm)	NSAIDs allowed	Headache frequency; VAS severity; disability	1 month	Significant reduction in headache frequency and severity (>50% responders)	Improvement less pronounced	rTMS superior to sham for migraine reduction	Mild drowsiness (n = 1)	Some concerns
Conforto et al., 2014 [27]	Brazil	Randomized, double-blind, proof-of-principle	18	9/9	38.8 ± 11.8	100%	Sham	Chronic migraine (ICHD); stable prophylaxis	Left DLPFC; 10 Hz; 1600 pulses/session; 23 sessions	Figure-of-eight (100 mm)	Restricted prophylaxis	Headache days; MIDAS	4 & 8 weeks	No significant reduction vs. baseline	Significant reduction in headache days and MIDAS	No superiority of rTMS over sham	None reported	Some concerns
Kumar et al., 2020 [24]	India	Randomized, double-blind, sham-controlled	20	10/10	33.5 ± 7.7	55%	Sham	Chronic migraine (ICHD-3)	Left DLPFC; 10 Hz; 70% RMT; 600 pulses/session; 10 sessions	Figure-of-eight	Preventive drugs allowed	Headache frequency; MIDAS	3 months	Significant reduction in headache frequency; no MIDAS difference	No improvement from baseline	rTMS reduced headache frequency but not disability	None reported	Some concerns
Brighina et al., 2004 [22]	Italy	Randomized, double-blind, sham-controlled	11	6/5	47 ± 7	63.6%	Sham	Chronic migraine (IHS); no depression	Left DLPFC; 20 Hz; 90% MT; 10 trains/session	Water-cooled figure-of-eight	Stable prophylaxis	Attack frequency; headache index; medication use	1–2 months	Significant and sustained reduction in all headache outcomes	No significant change	rTMS effective as adjunctive prophylaxis	None reported	Some concerns
Kalita et al., 2016 [25]	India	Randomized, double-blind, partially sham-controlled	98	52/46	31.8 ± 8.7	80.6%	Sham	≥4 attacks/month; no prophylaxis	Left frontal cortex; 10 Hz; 70% MT; 600 pulses/session; 3 sessions	Figure-of-eight (7 cm)	None	Headache frequency; VAS severity	1 month	78.6% achieved >50% VAS improvement	34.2% responders	rTMS superior to sham	None reported	High
Teepker et al., 2010 [19]	Germany	Randomized, double-blind, sham-controlled	27	14/13	35.5 ± 11	81.5%	Sham	≥4 migraine attacks/month	Vertex; 1 Hz; 1000 pulses/day; 5 days	Round (13 cm)	None	Migraine attacks; days; hours	8 weeks	Significant within-group improvement	No between-group difference	rTMS not superior to sham	Discomfort; dropouts	Some concerns

## Data Availability

Data sharing is not applicable.

## Supplementary Materials

The following supporting information can be downloaded at: https://www.mdpi.com/article/10.3390/neurolint18050080/s1, Table S1: Full Database Search Strings; Table S2: PRISMA Checklist.

## Abbreviations

The following abbreviations are used in this manuscript:

rTMS	Repetitive transcranial magnetic stimulation
MIDAS	Migraine Disability Assessment Questionnaire
ICHD	International Classification of Headache Disorders
RoB 2	Risk of Bias Tool 2
DLPFC	Dorsolateral prefrontal cortex
CGRP	Calcitonin gene-related peptide
EM	Episodic migraine
CM	Chronic migraine
VAS	Visual analogue scale
RMT	Resting motor threshold
MMD	Monthly migraine days
LTP	Long-term potentiation
LTD	Long-term depression
PAG	Periaqueductal gray

## References

[1] Burton W.N., Landy S.H., Downs K.E., Runken M.C. (2009). The Impact of Migraine and the Effect of Migraine Treatment on Workplace Productivity in the United States and Suggestions for Future Research. Mayo Clin. Proc..

[2] Olesen J. (2018). Headache Classification Committee of the International Headache Society (IHS) The International Classification of Headache Disorders, 3rd edition. Cephalalgia.

[3] Alkahtani R.F., Alrumaih S.S., Algezlan S.S., Almutairi R.R., Alturki B.A., Alanazi R.M., Alateeq F.A. (2022). The Impact of Migraine Disease on Work Productivity and Quality of Life Among the Adults in Riyadh, Saudi Arabia. Cureus.

[4] Lipton R.B., Stewart W.F., Von Korff M. (1997). Burden of migraine: Societal costs and therapeutic opportunities. Neurology.

[5] Bigal M.E., Liberman J.N., Lipton R.B. (2006). Age-dependent prevalence and clinical features of migraine. Neurology.

[6] Amiri P., Kazeminasab S., Nejadghaderi S.A., Mohammadinasab R., Pourfathi H., Araj-Khodaei M., Sullman M.J.M., Kolahi A.-A., Safiri S. (2022). Migraine: A Review on Its History, Global Epidemiology, Risk Factors, and Comorbidities. Front. Neurol..

[7] Bonafede M., Sapra S., Shah N., Tepper S., Cappell K., Desai P. (2018). Direct and Indirect Healthcare Resource Utilization and Costs Among Migraine Patients in the United States. Headache.

[8] Grazzi L. (2022). Mindfulness and other behavioral approaches. Neurol. Sci..

[9] Whyte C.A., Tepper S.J. (2009). Adverse effects of medications commonly used in the treatment of migraine. Expert. Rev. Neurother..

[10] Thorlund K., Toor K., Wu P., Chan K., Druyts E., Ramos E., Bhambri R., Donnet A., Stark R., Goadsby P.J. (2017). Comparative tolerability of treatments for acute migraine: A network meta-analysis. Cephalalgia.

[11] Ailani J., Lipton R.B., Goadsby P.J., Guo H., Miceli R., Severt L., Finnegan M., Trugman J.M. (2021). Atogepant for the Preventive Treatment of Migraine. N. Engl. J. Med..

[12] Dodick D.W., Turkel C.C., Degryse R.E., Aurora S.K., Silberstein S.D., Lipton R.B., Diener H.-C., Brin M.F. (2010). OnabotulinumtoxinA for treatment of chronic migraine: Pooled results from the double-blind, randomized, placebo-controlled phases of the PREEMPT clinical program. Headache.

[13] Edvinsson L., Haanes K.A., Warfvinge K., Krause D.N. (2018). CGRP as the target of new migraine therapies—Successful translation from bench to clinic. Nat. Rev. Neurol..

[14] Rossi S., Hallett M., Rossini P.M., Pascual-Leone A., Safety of TMS Consensus Group (2009). Safety, ethical considerations, and application guidelines for the use of transcranial magnetic stimulation in clinical practice and research. Clin. Neurophysiol..

[15] Lefaucheur J.P., André-Obadia N., Antal A., Ayache S.S., Baeken C., Benninger D.H., Cantello R.M., Cincotta M., de Carvalho M., De Ridder D. (2014). Evidence-based guidelines on the therapeutic use of repetitive transcranial magnetic stimulation (rTMS). Clin. Neurophysiol..

[16] Galhardoni R., Correia G.S., Araujo H., Yeng L.T., Fernandes D.T., Kaziyama H.H., Marcolin M.A., Bouhassira D., Teixeira M.J., de Andrade D.C. (2015). Repetitive transcranial magnetic stimulation in chronic pain: A review of the literature. Arch. Phys. Med. Rehabil..

[17] Lorenz J., Minoshima S., Casey K.L. (2003). Keeping pain out of mind: The role of the dorsolateral prefrontal cortex in pain modulation. Brain.

[18] Jiang Y., Yuan C., Sun P., Li C., Wang L. (2024). Efficacy and safety of high-frequency repetitive transcranial magnetic stimulation (rTMS) for migraine: A meta-analysis of randomized controlled trials. Acta Neurol. Belg..

[19] Teepker M., Hötzel J., Timmesfeld N., Reis J., Mylius V., Haag A., Oertel W., Rosenow F., Schepelmann K. (2010). Low-frequency rTMS of the vertex in the prophylactic treatment of migraine. Cephalalgia.

[20] Page M.J., McKenzie J.E., Bossuyt P.M., Boutron I., Hoffmann T.C., Mulrow C.D., Shamseer L., Tetzlaff J.M., Akl E.A., Brennan S.E. (2021). The PRISMA 2020 statement: An updated guideline for reporting systematic reviews. BMJ.

[21] Sterne J.A.C., Savović J., Page M.J., Elbers R.G., Blencowe N.S., Boutron I., Cates C.J., Cheng H.Y., Corbett M.S., Eldridge S.M. (2019). RoB 2: A revised tool for assessing risk of bias in randomised trials. BMJ.

[22] Brighina F., Piazza A., Vitello G., Aloisio A., Palermo A., Daniele O., Fierro B. (2004). rTMS of the prefrontal cortex in the treatment of chronic migraine: A pilot study. J. Neurol. Sci..

[23] Misra U.K., Kalita J., Bhoi S.K. (2013). High-rate repetitive transcranial magnetic stimulation in migraine prophylaxis: A randomized, placebo-controlled study. J. Neurol..

[24] Kumar A., Mattoo B., Bhatia R., Kumaran S., Bhatia R. (2021). Neuronavigation based 10 sessions of repetitive transcranial magnetic stimulation therapy in chronic migraine: An exploratory study. Neurol. Sci..

[25] Kalita J., Laskar S., Bhoi S.K., Misra U.K. (2016). Efficacy of single versus three sessions of high rate repetitive transcranial magnetic stimulation in chronic migraine and tension-type headache. J. Neurol..

[26] Song P., Li S., Shao Y., Zhu S., Wang Y., Xu P., Lin H. (2025). HF-rTMS of the left DLPFC relieve headaches and enhance frontal-temporal connectivity in migraine. Clin. Neurophysiol..

[27] Conforto A.B., Amaro E., Gonçalves A.L., Mercante J.P.P., Guendler V.Z., Ferreira J.R., Kirschner C.C., Peres M.F. (2014). Randomized, proof-of-principle clinical trial of active transcranial magnetic stimulation in chronic migraine. Cephalalgia.

[28] Vos T., Flaxman A.D., Naghavi M., Lozano R., Michaud C., Ezzati M., Shibuya K., Salomon J.A., Abdalla S., Aboyans V. (2012). Years lived with disability (YLDs) for 1160 sequelae of 289 diseases and injuries 1990-2010: A systematic analysis for the Global Burden of Disease Study 2010. Lancet.

[29] Siebner H.R., Funke K., Aberra A.S., Antal A., Bestmann S., Chen R., Classen J., Davare M., Di Lazzaro V., Fox P.T. (2022). Transcranial magnetic stimulation of the brain: What is stimulated?—A consensus and critical position paper. Clin. Neurophysiol..

