# Supplementary material for: Repetitive Transcranial Magnetic Stimulation in Migraine: Clinical Outcomes and Neurobiological Mechanisms—A Systematic Review
Source: Neurol Int. 2026 Apr 27;18(5):80. doi: 10.3390/neurolint18050080 (PMC13210037; doi:10.3390/neurolint18050080)
Supplement: Supplementary file 1 [file neurolint-18-00080-s001.zip › neurolint-4221711-supplementary table S1.pdf]

Supplementary Table S1: Full Database Search Strings

Note: Search strings were reconstructed from the original search protocol conducted from database inception to December 2025. Minor variations in syntax may exist across database-specific implementations. All searches were restricted to English-language publications and adult populations (≥18 years).

Database 1: PubMed (MEDLINE)

Date searched: December 2025

Search string:

("Transcranial Magnetic Stimulation"[MeSH Terms] OR "transcranial magnetic stimulation"[tiab] OR "repetitive transcranial magnetic stimulation"[tiab] OR "rTMS"[tiab] OR "TMS"[tiab]) AND ("Migraine Disorders"[MeSH Terms] OR "migraine"[tiab] OR "migraine with aura"[tiab] OR "migraine without aura"[tiab] OR "chronic migraine"[tiab] OR "episodic migraine"[tiab] OR "migraine headache"[tiab] OR "migraineur"[tiab]) AND ("randomized controlled trial"[pt] OR "randomized controlled trial"[tiab] OR "randomised controlled trial"[tiab] OR "RCT"[tiab] OR "sham"[tiab] OR "placebo"[tiab] OR "controlled trial"[tiab])

Filters applied: English language; Adult: 18+ years

Records retrieved: 28

Database 2: PsycNet (APA PsycINFO)

Date searched: December 2025

Search string: (DE "Transcranial Magnetic Stimulation" OR AB "transcranial magnetic stimulation" OR AB "repetitive transcranial magnetic stimulation" OR AB "rTMS") AND (DE "Migraines" OR AB "migraine" OR AB "migraine headache" OR AB "chronic migraine" OR AB "episodic migraine" OR AB "migraine with aura" OR AB "migraine without aura") AND (AB "randomized controlled trial" OR AB "randomized controlled trial" OR AB "sham" OR AB "placebo" OR AB "controlled trial")

Filters applied: English language; Peer-reviewed journals; Population: Adulthood (18 yrs & older)

Records retrieved: 11

Database 3: Ovid MEDLINE

Date searched: December 2025

Search string:

1. exp Transcranial Magnetic Stimulation/
2. (transcranial magnetic stimulation or repetitive transcranial magnetic stimulation or rTMS or TMS).ti,ab.
3. 1 OR 2

4. exp Migraine Disorders/
5. (migraine or migraine with aura or migraine without aura or chronic migraine or episodic migraine or migraine headache or migraineur).ti,ab.
6. 4 OR 5

7. exp Randomized Controlled Trials as Topic/
8. randomized controlled trial.pt.
9. (randomized or randomised or sham or placebo or controlled trial).ti,ab.
10. 7 OR 8 OR 9

11. 3 AND 6 AND 10

Filters applied: English language; Humans; Adult (18+ years)

Records retrieved: 14

Database 4: Ovid Embase

Date searched: December 2025

Search string:

1. exp transcranial magnetic stimulation/
2. (transcranial magnetic stimulation or repetitive transcranial magnetic stimulation or rTMS or TMS).ti,ab.
3. 1 OR 2

4. exp migraine/
5. (migraine or migraine with aura or migraine without aura or chronic migraine or episodic migraine or migraine headache or migraineur).ti,ab.
6. 4 OR 5

7. exp randomized controlled trial/
8. (randomized or randomised or sham or placebo or controlled trial).ti,ab.
9. 7 OR 8

10. 3 AND 6 AND 9

Filters applied: English language; Human; Adult (18+ years)

Records retrieved: 8

Total records across all databases (before deduplication): 61

Duplicates removed: 20

Records screened: 41
